# A simple technique to aid intraoperative identification of the tibial mechanical axis during total knee replacement

**DOI:** 10.1308/003588413X13511609957056j

**Published:** 2013-01

**Authors:** N Frew, P Loughenbury, B Hopton

**Affiliations:** ^1^Yorkshire and the Humber Deanery, UK; ^2^Airedale NHS Foundation Trust, UK

## Background

Successful total knee replacement (TKR) requires accurate component placement and coronal plane malalignment of greater than 3º varus/valgus from the mechanical axis has been shown to predispose to early failure.^1–3^ An extramedullary alignment jig is most commonly used to identify the mechanical axis. The distal end of the jig should be centred over the ankle mortise, which can be difficult to assess when the leg is covered by surgical drapes, particularly in obese patients.

## Technique

**Figure 1:**
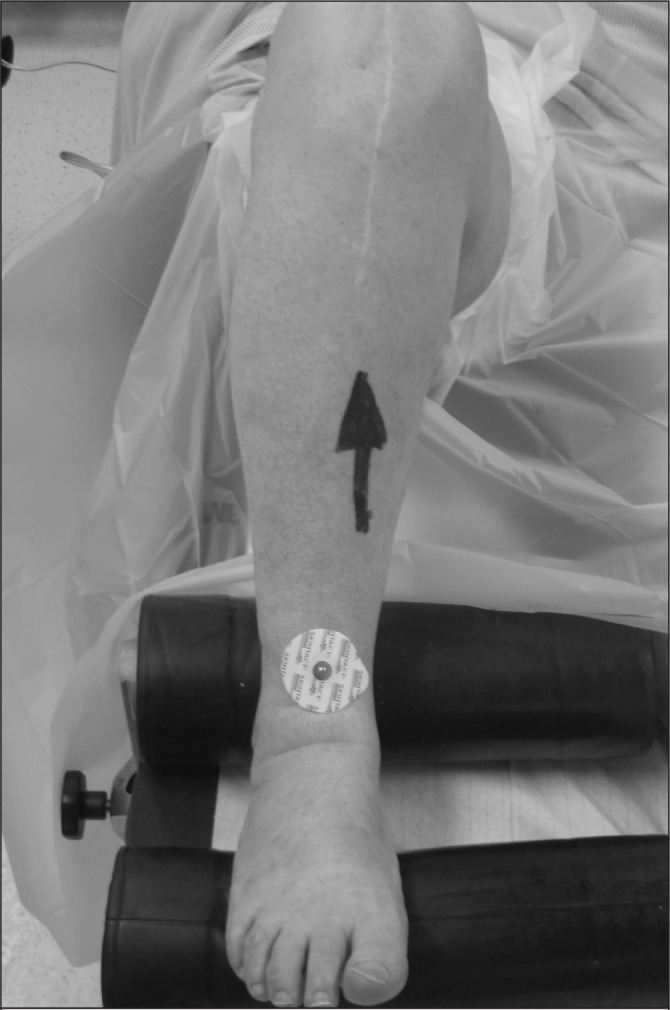
Patient positioned preoperatively with an electrocardiography electrode placed over the extensor hallucis longus tendon

Preoperatively, the centre of the distal tibia is identified and an electrocardiography (ECG) electrode is placed on the overlying skin (Fig 1). There is no consensus in the literature as to the most accurate method for identifying this point. Several landmarks are commonly used including the extensor hallucis longus tendon,^4^ the tibialis anterior tendon^5^ and a point 3–5 mm medial to the midpoint of the malleoli.^6^ Intraoperatively, the ECG electrode is easily palpable through the surgical drapes and greatly assists accurate distal positioning of the tibial alignment jig.

## Discussion

Several studies have shown that using standard techniques, postoperative TKR alignment lies outside the desired ±3º varus/valgus range in up to 30% of cases.^7,8^ Computer navigation has been shown to improve alignment but has failed to gain widespread acceptance owing to factors such as additional cost and surgical time. Our simple technique provides an easily palpable distal reference for determining tibial alignment using an ECG electrode, which can be readily found in any anaesthetic room.
